# The prognostic value of neuromedin U in patients with hepatocellular carcinoma

**DOI:** 10.1186/s12885-020-6532-1

**Published:** 2020-02-03

**Authors:** Qiao Li, Lingyu Han, Shengnan Ruan, Shunli Shen, Qinghua Cao, Xiuqin Cai, Yuan Yan, Baogang Peng, Yunpeng Hua

**Affiliations:** 1 Department of Liver Surgery, the First Affiliated Hospital of Sun Yat-sen University, Guangzhou, Guangdong 510080 People’s Republic of China; 2 Department of Colorectal Surgery, Fudan University Shanghai Cancer Center, Shanghai, 200032 People’s Republic of China; 3 Department of Pathology, the First Affiliated Hospital of Sun Yat-sen University, Guangzhou, Guangdong 510080 People’s Republic of China; 4 Department of Gastrointestinal Surgery, the First Affiliated Hospital of Sun Yat-sen University, Guangzhou, Guangdong 510080 People’s Republic of China; 5 Department of Histology and Embryology, College of Basic Medicine, Southern Medical University, Guangzhou, Guangdong 510515 People’s Republic of China

**Keywords:** Hepatocellular carcinoma, Neuromedin U, Prognosis, Cytokine, Macrophage

## Abstract

**Background:**

Neuromedin U (NMU) is a neuropeptide belonging to the neuromedin family. Recently, significant associations between NMU and several cancers have been reported. However, no studies have examined the association between NMU and hepatocellular carcinoma (HCC). The purpose of this study was to examine the role of NMU in HCC.

**Methods:**

An enzyme-linked immunosorbent assay was used to measure the level of NMU protein in the sera of patients with hepatic hemangioma and HCC. NMU and cytokine mRNA expression was assessed in HCC samples via RT-qPCR. A tissue microarray consisting of 228 HCC peri- and intra-tumor tissues was used to detect NMU expression via immunohistochemical analysis. The association between NMU expression and overall survival (OS) and disease-free survival (DFS) was analyzed by Kaplan-Meier curves, the log-rank test, and Cox proportional hazard model.

**Results:**

The level of NMU protein was increased in the sera of HCC patients (*p* = 0.006). NMU was expressed in intercellular space, rather than in hepatocytes or HCC cells. The prognosis of HCC patients with high NMU expression in peri-tumor tissue was significantly poorer than that of patients with low NMU expression (OS: *p* = 0.002, DFS: *p* = 0.033). Peri-tumor NMU expression was also a significant independent prognostic factor for OS (hazard ratio: 1.541, 95% confidence interval: 1.092–2.175, *p* = 0.014). The level of NMU expression was positively associated with M2 macrophage percentage and the levels of type-2 inflammatory cytokines in HCC tissue.

**Conclusions:**

NMU may serve as a novel prognostic biomarker for HCC patients, although further validation is needed in the future. The activation of M2 macrophages and a type-2 inflammatory response may involve in the role of NMU in patients with HCC.

## Background

As a frequent and aggressive cancer, hepatocellular carcinoma (HCC) is now the fourth most common cause of cancer-related deaths worldwide, from which 781,631 patients die in 2018 [1, 2]. There are a number of reasons for the poor outcomes of HCC patients. First, most patients with HCC are usually diagnosed at an advanced stage, and lose the opportunity of curative hepatectomy. Second, even if a radical operation is performed, the rate of recurrence and metastasis is still as high as 60 to 70% within 5 years [3–5]. Thus, there is an urgent need to find the novel and effective diagnostic markers for early HCC, and new therapeutic strategies to improve the outcomes of patients with HCC.

Neuromedin U (NMU) is a neuropeptide expressed in various organs and tissues, with the strongest expression in the gastrointestinal tract and central nervous system [6–8]. The neuropeptide has been demonstrated to be associated with a range of different physiological functions, including smooth muscle contraction, energy homeostasis, nociception, circadian control, bone remodeling, and immune regulation [9–11]. A limited number of studies had suggested that NMU may play an oncogenic role in the progression of various cancers, including lung cancer, pancreatic cancer, breast cancer, renal cancer, and endometrioid endometrial carcinoma, through promoting migration, invasion, glycolysis, a mesenchymal phenotype, a stem cell phenotype of cancer cells, and resistance to the anti-tumor immune response [12–19]. However, besides a report of NMU exacerbating non-alcoholic steatohepatitis (NASH) [20], the effects of NMU on liver diseases, especially on HCC, have not yet been studied.

In the present study, we investigated the value of NMU as a prognostic marker for patients with HCC by detecting NMU expression in 228 HCC peri- and intra-tumor tissues. Then, we further studied the correlation of the level of NMU expression with the percentage of M2 macrophages or the expression of type-2 inflammatory cytokines in peri-tumor and intra-tumor tissues.

## Methods

### Patients and tissue samples

A total of 228 patients who received a hepatectomy as first-line treatment for HCC from January 2006 to September 2009 at The First Affiliated Hospital of Sun Yat-sen University (Guangzhou, China) were enrolled in this study. The majority of patients (201/228) were infected by hepatitis B virus (HBV). Two patients were infected by hepatitis C virus (HCV) and 2 patients are with HCC family history without HBV and HCV. Patients who met all of the following criteria were included in this study: (1) no previous treatment for HCC before surgery; (2) histologic confirmation of HCC; (3) R0 resection. Informed written consent was obtained from all patients. The present study was approved by the First Affiliated Hospital of Sun Yat-sen University Ethics Committee.

The preoperative diagnosis of HCC was based on the criteria of the American Association for the study of Liver Disease [2]. The volume of liver resection, and the surgical procedure was decided by tumor size, tumor location, and liver functional reserve according to a multidisciplinary team meeting every week. Tumor stages were classified according to the 8th edition of the tumor-node- metastasis (TNM) staging system of the American Joint Committee on Cancer (AJCC) [21]. Fresh HCC intra-tumor and peri-tumor liver tissues were collected within 30 min after resection. These tissues were fixed with 10% formalin, embedded in paraffin, and then made into a tissue microarray.

### Immunohistochemistry

The immunohistochemistry techniques used in this study have been described previously [22]. Tissue microarrays and sections were used to examine the level of NMU expression and the number of M2 macrophages in peri-tumor and intra-tumor tissue. The tissue sections were incubated with primary rabbit anti-NMU antibody (Abcam, 1:50), primary mouse anti-CD68 antibody (Abcam, 1:400), or primary rabbit anti-CD206 antibody (Abcam, 1:2000) at 4 °C overnight. Negative controls were treated in the same manner, omitting the primary antibodies. A Dako Real Envision Detection System (Dako) was then used for visualization.

### Evaluation of immunohistochemical staining

Images were obtained with an Olympus BX63 microscope and ZEISS Axio Scan.Z1 Digital Slide Scanner. The immunohistochemical staining in the tissue was scored independently by 2 pathologists blinded to the clinical data. The number of CD68 and C206 positive-stained cells was determined and quantified using the Image Scope positive pixel count algorithm (Aperio) [23].

NMU staining was scored by applying a semiquantitative immunoreactivity score (IRS) reported elsewhere [24]. Category A documented the intensity of immunostaining as 0–3 (0, negative; 1, weak; 2, moderate; 3, strong). Category B documented the percentage of immunoreactive cells as 0 (< 5%), 1 (6 to 25%), 2 (26 to 50%), 3 (51 to 75%), and 4 (76 to 100%). Multiplication of category A and B results gave an IRS ranging from 0 to 12. Sections with a total score of 0 or 1 or 2 were defined as negative (−), with a total score of 3 or 4 were defined as weakly positive (+), with a total score of 6 or 8 were defined as moderately positive (++), and those with a total score of 9 or 12 were defined as strongly positive (+++). Peri- and intra-tumor tissue in each case was classified as high or low NMU expression, as determined by receiver operating characteristic (ROC) curve analysis [23]. For categorical analyses, the immunoreactivity was graded as low level (total score < =4) or high level (total score > 4) in peri- and intra-tumor tissue.

### Enzyme-linked immunosorbent assay (ELISA)

Blood samples from 10 hepatic hemangioma (HH) patients and 10 patients with HCC were collected, and the serum was isolated. Blood samples were allowed to clot for 2 h at room temperature or overnight at 4 °C, and then were centrifuged for 20 min at approximately 1000×*g* to obtain serum. NMU protein was then detected in the serum using an ELISA kit (Cloud-Clone Corp.), according to the manufacturer’s instructions.

### Quantitative real-time PCR

Total RNA was extracted from frozen peri-tumor tissue (*n* = 12) and intra-tumor tissue (n = 12) using TRIzol reagent (Invitrogen). Complementary DNA (cDNA) was synthesized using the Prime Script RT Reagent Kit Perfect Real-Time Kit (TaKaRa Bio Inc.), and then used for quantitative real-time PCR (RT-qPCR) using SYBR PremixEx Taq (TaKaRa Bio Inc.). The relative expression levels of mRNAs were calculated by the 2-ΔCt method. The primers used for the amplification of human genes were as follows:

NMU forward: 5′- AGT TGT GGA ATG AGG CAT CC − 3′, reverse: 5′- GGA TGC ACA ACT GAC GAC AC − 3′. Interleukin (IL)-6 forward: 5′- TCA ATA TTA GAG TCT CAA CCC CCA − 3′, reverse: 5′- GAA GGC GCT TGT GGA GAA GG -3′. IL-8 forward: 5′- CAC CGG AAG GAA CCA TCT CA − 3′, reverse: 5′- TGG CAA AAC TGC ACC TTC ACA − 3′. Tumor necrosis factor (TNF)-α forward: 5′- CGA GTG ACA AGC CTG TAG C − 3′, reverse: 5′- GGT GTG GGT GAG GAG CAC AT -3′. IL-4 forward: 5′- AGG AAG CCA ACC AGA GTA − 3′, reverse: 5′- CGA ACA CTT TGA ATA TTT CTC TCT -3′. IL-10 forward: 5′- GAT CCA GTT TTA CCT GGA GGA − 3′, reverse: 5′- CCT GAG GGT CTT CAG GTT CTC -3′. IL-13: forward 5′- ATG GTA TGG AGC ATC AAC − 3′, reverse: 5′- CAT CCT CTG GGT CTT CTC − 3′. β-actin: forward 5′- GCA CTC TTC CAG CCT TCC TT − 3′, reverse: 5′- GTT GGC GTA CAG GTC TTT GC − 3′.

### Follow-up

After surgery, patients were followed-up once a month for the first 6 months, and then every 3 months thereafter. Serum alpha-fetoprotein (AFP) and abdominal ultrasonography were done routinely at the postoperative visits. Computed tomography (CT) or magnetic resonance imaging (MRI) was performed every 3 to 6 months. Once tumor recurrence was verified by these examinations, patients received optimal treatment, including second hepatectomy, radiofrequency ablation (RFA), transcatheter arterial chemoembolization (TACE), or targeted therapy. Death of patients was determined from death certificates or phone follow-up.

### Statistical analysis

Data were presented as mean ± standard deviation. All analyses were performed with GraphPad Prism 5.0 and SPSS 19.0 software. Comparisons and correlations of quantitative data between 2 groups were performed by unpaired Student’s t test and chi-square test, respectively. Categorical data were analyzed by Fisher’s exact test. The Cox proportional hazard model was used to assess the prognostic values of the variables. Clinical variables that had significant prognostic value in univariate analysis were subsequently included in a multivariate analysis. The overall survival (OS) and disease-free survival (DFS) rates were calculated by the Kaplan-Meier method, and survival curves were compared by log-rank test. All statistical tests were 2-sided, and a significant difference was considered when a value of *p* was < 0.05.

## Results

### The level of NMU in the serum and tissues of HCC patients

**Fig. 1:**
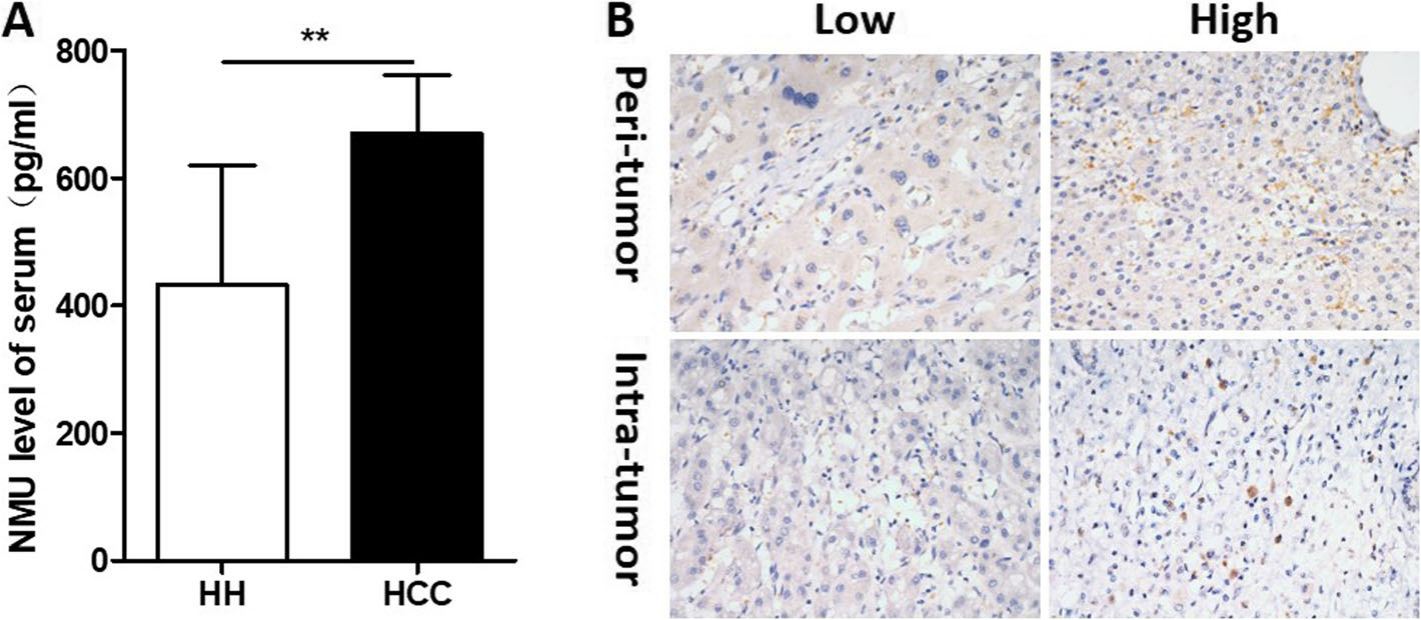
IL-25 is highly expressed in HCC patients. **a** The level of NMU were detected by ELISA in serum of hepatic hemangioma patients (*n* = 10) and HCC patients (*n* = 10). **b** Immunohistochemistry (IHC) staining was performed in a tissue microarray consisted of 228 HCC peri- and intra-tumor tissue, NMU representative IHC images are shown. Bar, 20 μm. ** *p* < 0.01. NMU, neuromedin U; HH, hepatic hemangioma; HCC, hepatocellular carcinoma

The level of NMU in the serum of HCC patients was significantly greater than in HH (non-cancer) patients (*p* = 0.006) (Fig. 1a). Immunohistochemical staining showed that NMU protein was present in intercellular space of HCC peri- and intra-tumor tissue, rather in hepatic cells or HCC cells (Fig. 1b). These data suggested that NMU expression was upregulated in HCC patients, and it was mainly distributed in intercellular space.

### High expression of NMU in peri-tumor tissue predicts a poor prognosis in patients with HCC

**Fig. 2:**
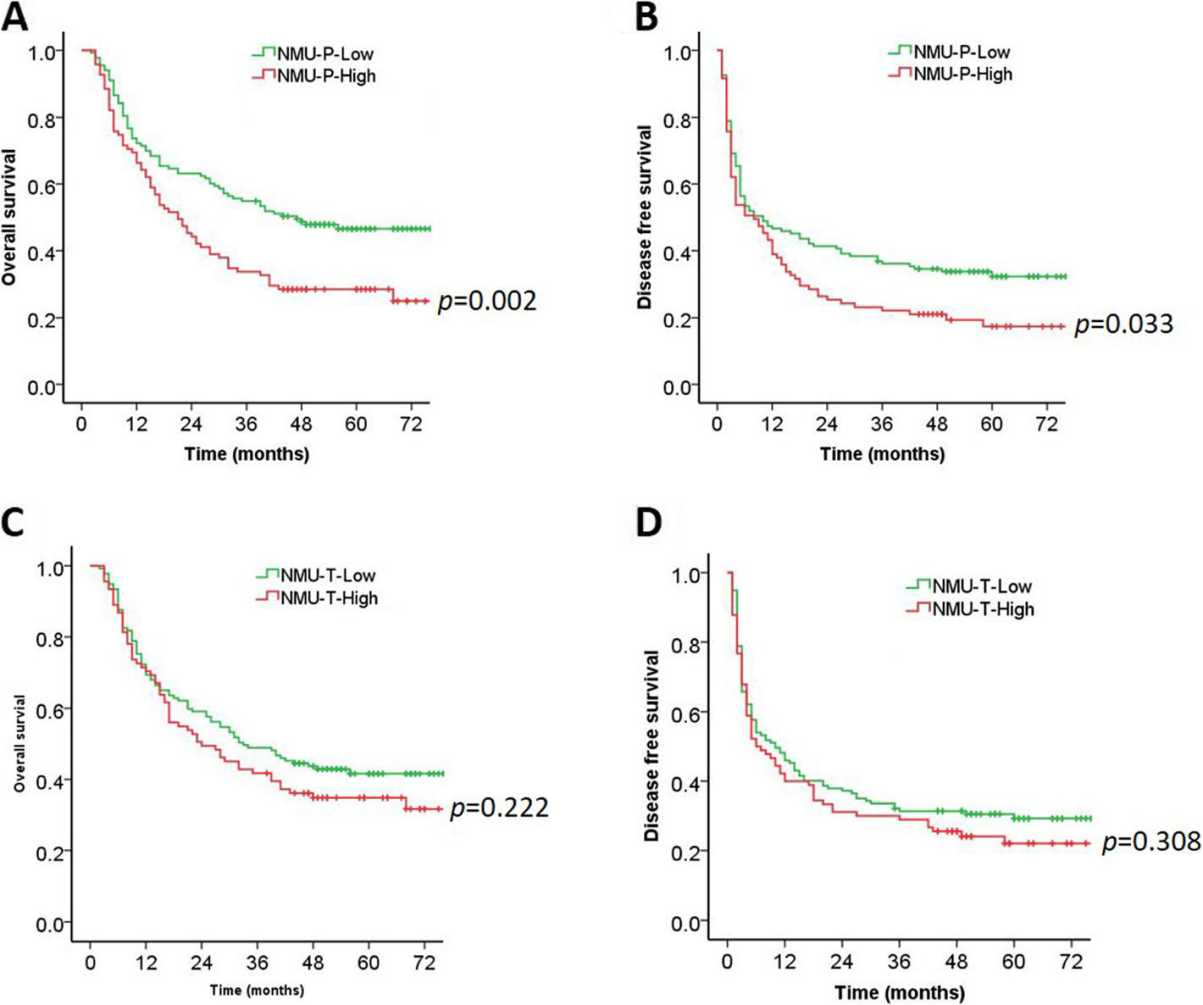
Kaplan-Meier curves are shown for time to OS and DFS among patients with high or low peri- and intra-tumor NMU expression. **a, b** OS and DFS curves for HCC patient groups with low (*n* = 133) and high (*n* = 95) NMU expression in peri-tumor tissue. **c, d** OS and DFS curves of HCC patients in correlation with NMU expression in intra-tumor tissue, the patients with HCC were divided into 2 groups: low (*n* = 137), high (*n* = 91). NMU, neuromedin U; P, peri-tumor; T, intra-tumor

The 228 patients with HCC were divided into 2 groups according to the NMU expression in peri-tumor tissue: low NMU expression group (*n* = 133) and high NMU expression group (*n* = 95) (Table 1). Kaplan-Meier survival analysis indicated that high expression of NMU in peri-tumor tissue was positively correlated with poor survival after resection in HCC patients (Fig. 2a, b). The 1-, 3-, and 5-year OS rates of the NMU high group were significantly lower than that of the NMU low group (66.3, 33.7, and 28.4% vs. 72.2, 54.9, and 46.6%, respectively, *p* = 0.002) (Fig. 2a, Table 2). The 1-, 3-, and 5-year DFS rates of the NMU high group were also markedly lower than those of the NMU low group (38.9, 22.1, and 17.4% vs. 46.6, 36.1, and 32.2%, respectively, *p* = 0.033) (Fig. 2b, Table 2).

**Table 1:** The expression of NMU in HCC

Group	*n*	Expression of NMU	
		Low	High
Peri-tumor	228	133 (58.3%)	95 (41.7%)
Intra-tumor	228	137 (60.1%)	91 (39.9%)

**Table 2:** Overall and disease-free survival based on the NMU expression of peri- and intra-tumor tissue

Group	case	Overall survival			Disease-free survival		
		1 year (%)	3 years (%)	5 years (%)	1 year (%)	3 years (%)	5 years (%)
All	228	69.7	46.1	38.9	43.4	30.3	26.2
Peri-tumor							
Low	133	72.2	54.9	46.6	46.6	36.1	32.2
High	95	66.3	33.7	28.4	38.9	22.1	17.4
Intra-tumor							
Low	137	69.3	48.9	41.6	48.2	31.4	29.3
High	91	70.3	41.8	34.8	42.2	28.9	22.0

The 228 HCC patients were also divided into 2 groups according to NMU expression in intra-tumor tissue: low NMU expression group (*n* = 137) and high NMU expression group (*n* = 91) (Table 1). The 3-, and 5-year OS rates of the NMU high group (41.8 and 34.8%) were lower than those of the NMU low group (48.9 and 41.6%), although there was no significance (*p* = 0.222) (Fig. 2c and Table 2). Likewise, the 1-, 3-, and 5-year DFS rates of NMU high group were lower than that of the NMU low group (42.2, 28.9, and 22% vs. 48.2, 31.4, and 29.3%, respectively, *p* = 0.308) (Fig. 2d and Table 2).

### Relationship between NMU expression and 16 clinicopathological features

To further understand the prognostic value of NMU expression after HCC resection, we analyzed the associations of NMU expression in peri- and intra-tumor tissue with 16 clinicopathological features, including sex, age, HCC family history, hepatitis B surface antigen (HBsAg), cirrhosis, tumor number, tumor size, major vascular invasion, Edmondson grade, TNM stage, Child-Pugh class, platelet (PLT) count, total bilirubin (TBIL) level, alanine aminotransferase (ALT) level, aspartate aminotransferase (AST) level, and AFP level. High NMU expression in peri-tumor tissue was positively correlated with a high level of AST (*p* = 0.031) (Table 3). There was no significant relationship between the expression of NMU in intra-tumor tissue and the 16 clinicopathological features (Table 3).

**Table 3:** Relationship between NMU expression and 16 clinicopathological features

Variables	Cases	NMU expression (peri-tumor)		*P* value	NMU expression (intra-tumor)		*P* value
		Low	High		Low	High	
Sex							
Male	203	121(59.6%)	82(40.4%)	0.267	123(60.6%)	80(39.4%)	0.658
Female	25	12(48.0%)	13(52.0%)		14(56.0%)	11(44.0%)	
Age (yrs)							
≤ 60	179	106(59.2%)	73(40.8%)	0.605	111(62%)	68(38%)	0.257
>60	49	27(55.1%)	22(44.9%)		26(53.1%)	23(46.9%)	
HCC family history							
Yes	13	10(76.9%)	3(23.1%)	0.162	10(76.9%)	3(23.1%)	0.202
No	215	123(57.2%)	92(42.8%)		127(59.1%)	88(40.9%)	
HBsAg							
Positive	201	121(60.2%)	80(39.8%)	0.119	119(59.2%)	82(40.8%)	0.457
Negative	27	12(44.4%)	15(55.6%)		18(66.7%)	9(33.3%)	
Cirrhosis							
Yes	180	107(59.4%)	73(40.6%)	0.510	108(60.0%)	72(40.0%)	0.958
No	48	26(54.2%)	22(45.8%)		29(60.4%)	19(39.6%)	
Tumor number							
Single	161	90(55.9%)	71(44.1%)	0.228	92(57.1%)	69(42.9%)	0.159
Multiple	67	43(64.2%)	24(35.8%)		45(67.2%)	22(32.8%)	
Tumor size (cm)							
≤ 5	81	50(61.7%)	31(38.3%)	0.440	42(51.9%)	39(48.1%)	0.059
>5	147	83(56.5%)	64(43.5%)		95(64.6%)	52(35.4%)	
Major vascular invasion							
Yes	42	23(54.8%)	19(45.2%)	0.603	28(66.7%)	14(33.3%)	0.335
No	186	110(59.1%)	76(40.9%)		109(58.6%)	77(41.4%)	
Edmondson grade							
I-II	177	101(57.1%)	76(42.9%)	0.468	105(59.3%)	72(40.7%)	0.660
III-IV	51	32(62.7%)	19(37.3%)		32(62.7%)	19(37.3%)	
TNM stage							
I-II	151	88(58.3%)	63(41.7%)	0.981	87(57.6%)	64(42.4%)	0.286
III-IV	77	45(58.4%)	32(41.6%)		50(64.9%)	2735.1%)	
Child-Pugh class							
A	210	125(59.5%)	85(40.5%)	0.213	127(60.5%)	83(39.5%)	0.682
B	18	8(44.4%)	10(55.6%)		10(55.6%)	8(44.2%)	
PLT count (× 10^9^)							
≥ 100	206	120(58.3%)	86(41.7%)	0.940	123(59.7%)	83(40.3%)	0.721
<100	22	13(59.1%)	9(40.9%)		14(63.6%)	8(36.4%)	
TBIL (μmol/L)							
<34.2	210	126(60.0%)	84(40.0%)	0.081	129(61.4%)	81(38.6%)	0.158
≥ 34.2	18	7(38.9%)	11(61.1%)		8(44.4%)	10(55.6%)	
ALT (U/L)							
≤ 40	190	115(60.5%)	75(39.5%)	0.133	111(58.4%)	79(41.6%)	0.251
>40	38	18(47.4%)	20(52.6%)		26(68.4%)	12(31.6%)	
AST (U/L)							
≤37	179	111(62.0%)	68(38.0%)	**0.031**	106(59.2%)	73(40.8%)	0.608
>37	49	22(44.2%)	27(55.1%)		31(63.3%)	18(36.7%)	
AFP (ug/L)							
≤ 20	55	29(52.7%)	26(47.3%)	0.333	32(58.2%)	23(41.8%)	0.740
>20	173	104(60.1%)	69(39.9%)		105(60.7%)	68(39.3%)	

Abbreviations: *HCC* hepatocellular carcinoma, *HBsAg* hepatitis B surface antigen, *TNM* tumor-node- metastasis, *PLT* platelet, *TBIL* total bilirubin, *ALT* alanine aminotransferase, *AST* aspartate aminotransferase, *AFP* a-fetoprotein. It was considered to have significance when a value of *p* was < 0.05

### NMU in peri-tumor is an independent prognostic marker in patients with HCC

To further identify risk factors associated with OS and DFS, NMU expression and 16 clinicopathological features were evaluated by univariate analysis and the Cox proportional hazard regression model. Univariate analysis indicated that peri-tumor tissue NMU expression, tumor number, tumor size, major vascular invasion, Edmondson grade, TNM stage, Child-Pugh class, PLT count, and ALT and AST level were significant prognostic factors for DFS and OS (Tables 4 and 5). Furthermore, Cox regression multivariate analysis indicated that tumor size (*p* = 0.022), Edmondson grade (*p* = 0.020), TNM stage (*p <* 0.001), Child-Pugh class (*p* = 0.028), and AST level (*p =* 0.002) were significant independent prognostic factors for DFS (Table 4). In addition, Edmondson grade (*p* = 0.005), TNM stage (*p <* 0.001), AST level (*p <* 0.001) and peri-tumor tissue NMU expression (*p* = 0.014) was found to be a significant independent prognostic factors for OS (Table 5).

**Table 4:** Univariate and multivariate analyses of clinicopathologic parameters associated with disease-free survival

Clinical parameters	Univariate analysis			Multivariate analysis		
	HR	95% CI	*P* value	HR	95% CI	*P* value
Sex (male vs. female)	0.914	0.560–1.492	0.720			
Age (yrs) (≤60 vs. >60)	1.092	0.765–1.558	0.630			
HCC family history (yes vs. no)	1.218	0.642–2.309	0.546			
HBsAg (positive vs. negative)	0.703	0.425–1.162	0.169			
Cirrhosis (yes vs. no)	0.916	0.635–1.320	0.636			
Tumor number (single vs. multiple)	2.163	1.569–2.981	**<0.001**			
Tumor size (cm) (≤5 vs. >5)	2.355	1.670–3.320	**<0.001**	1.551	1.065–2.258	**0.022**
Major vascular invasion (yes vs. no)	2.510	1.743–3.617	**<0.001**			
Edmondson grade (I + II vs. III + IV)	1.584	1.118–2.244	**0.010**	1.514	1.067–2.149	**0.020**
TNM stage (I + II vs. III + IV)	3.077	2.240–4.225	**<0.001**	2.484	1.767–3.494	**<0.001**
Child-Pugh class (A vs. B)	1.846	1.112–3.063	**0.018**	1.789	1.064–3.008	**0.028**
PLT count (×10^9^) (≥100 vs. <100)	2.623	1.338–5.141	**0.005**			
TBIL (μmol/L) (<34.2 vs. ≥34.2)	1.373	0.819–2.301	0.229			
ALT (U/L) (≤40 vs. >40)	1.281	0.867–1.894	0.214			
AST (U/L) (≤37 vs. >37)	2.328	1.643–3.299	**<0.001**	1.775	1.225–2.572	**0.002**
AFP (ug/L) (≤20 vs. >20)	1.218	0.850–1.744	0.282			
NMU peri-tumor (low vs. high group)	1.374	1.012–1.865	**0.041**			
NMU intra-tumor (low vs. high group)	1.179	0.867–1.604	0.294			

Abbreviations: *HR* hazard ratio, *CI* confidence interval, *HCC* hepatocellular carcinoma, *HBsAg* hepatitis B surface antigen, *TNM* tumor-node- metastasis, *PLT* platelet, *TBIL* total bilirubin, *ALT* alanine aminotransferase, *AST* aspartate aminotransferase, *AFP* a-fetoprotein, *NMU* neuromedin U. It was considered to have significance when a value of *p* was < 0.05

**Table 5:** Univariate and multivariate analyses of clinicopathologic parameters associated with overall survival

Clinical parameters	Univariate analysis			Multivariate analysis		
	HR	95% CI	*P* value	HR	95% CI	*P* value
Sex (male vs. female)	0.726	0.410–1.285	0.271			
Age (yrs) (≤60 vs. >60)	0.991	0.661–1.484	0.964			
HCC family history (yes vs. no)	0.710	0.313–1.609	0.412			
HBsAg (positive vs. negative)	0.906	0.538–1.525	0.710			
Cirrhosis (yes vs. no)	1.055	0.704–1.581	0.795			
Tumor number (single vs. multiple)	2.545	1.808–3.582	**<0.001**			
Tumor size (cm) (≤5 vs. >5)	2.818	1.894–4.192	**<0.001**			
Major vascular invasion (yes vs. no)	2.869	1.967–4.184	**<0.001**			
Edmondson grade (I + II vs. III + IV)	1.711	1.181–2.479	**0.005**	1.718	1.179–2.504	**0.005**
TNM stage (I + II vs. III + IV)	3.851	2.739–5.413	**<0.001**	3.303	2.320–4.702	**<0.001**
Child-Pugh class (A vs. B)	1.813	1.041–3.159	**0.036**			
PLT count (×10^9^) (≥100 vs. <100)	2.254	1.103–4.603	**0.026**			
TBIL (μmol/L) (<34.2 vs. ≥34.2)	1.394	0.787–2.470	0.255			
ALT (U/L) (≤40 vs. >40)	1.517	1.001–2.299	**0.049**			
AST (U/L) (≤37 vs. >37)	3.265	2.261–4.716	**<0.001**	2.407	1.628–3.559	**<0.001**
AFP (ug/L) (≤20 vs. >20)	1.329	0.887–1.992	0.168			
NMU peri-tumor (low vs. high group)	1.691	1.211–2.361	**0.002**	1.541	1.092–2.175	**0.014**
NMU intra-tumor (low vs. high group)	1.229	0.879–1.720	0.228			

Abbreviations: *HR* hazard ratio, *CI* confidence interval, *HCC* hepatocellular carcinoma, *HBsAg* hepatitis B surface antigen, *TNM* tumor-node- metastasis, *PLT* platelet, *TBIL* total bilirubin, *ALT* alanine aminotransferase, *AST* aspartate aminotransferase, *AFP* a-fetoprotein, *NMU* neuromedin U. It was considered to have significance when a value of *p* was < 0.05

### NMU expression is associated with the level of M2 macrophages and type-2 immune response factors

**Fig. 3:**
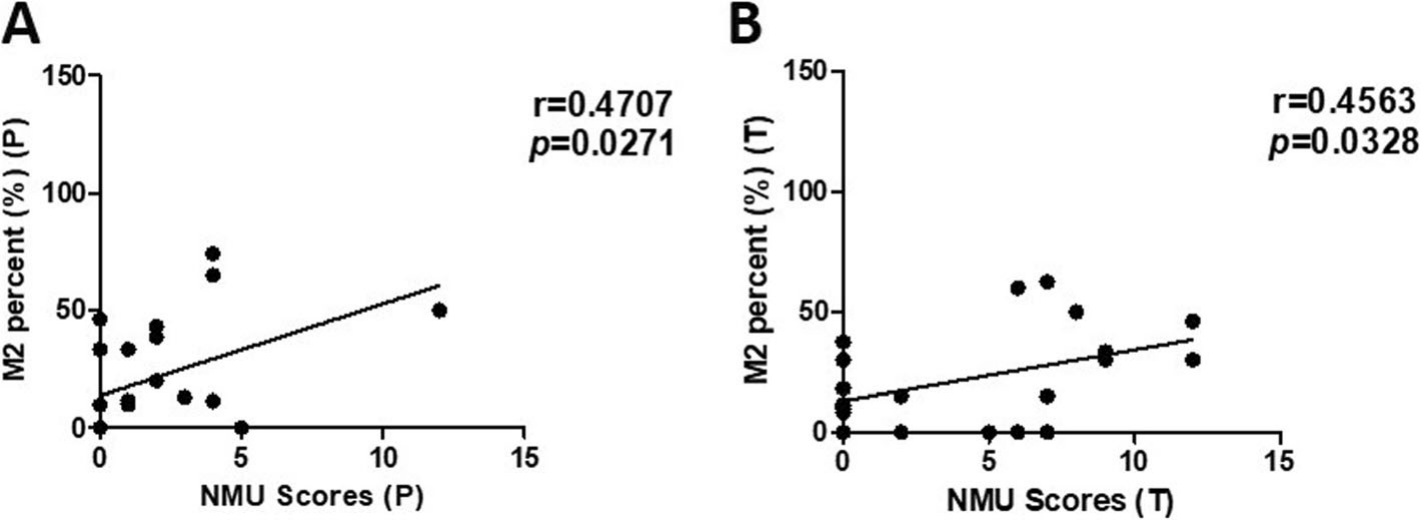
Correlation between NMU expression and M2 macrophage percentage. **a-b** Immunohistochemistry staining was performed on 22 HCC peri- and intra-tumor tissue. Correlation between M2 macrophage (CD206/CD68) percentage and NMU expression in peri-tumor **a** and intra-tumor **b** tissue. NMU, neuromedin U; M2, type-2 macrophage; P, peri-tumor; T, intra-tumor

Studies have shown that NMU can rapidly promote type-2 cytokine responses via activating group 2 innate lymphoid cells [6, 7, 25]. Moreover, Teranishi et al. [20] reported that NMU-immunoreactive cells were present in the macrophage population of livers with non-alcoholic steatohepatitis (NASH). This is consistent with our findings that histochemical localization of NMU was in intercellular space of HCC peri- and intra-tumor tissue. Thus, we hypothesized that the effect of NMU on HCC was related to activation of M2 macrophages and promotion of type-2 cytokine responses. Thus, we examined the M2 macrophage percentage (CD206/CD68) in HCC peri- and intra-tumor tissue using immunohistochemical staining. We found a significant positive correlation between NMU level and M2 percentage (CD206/CD68) in HCC peri- (r = 0.4624, *p* = 0.0302) and intra-tumor (r = 0.4448, *p* = 0.0381) tissue (Fig. 3a, b).

**Fig. 4:**
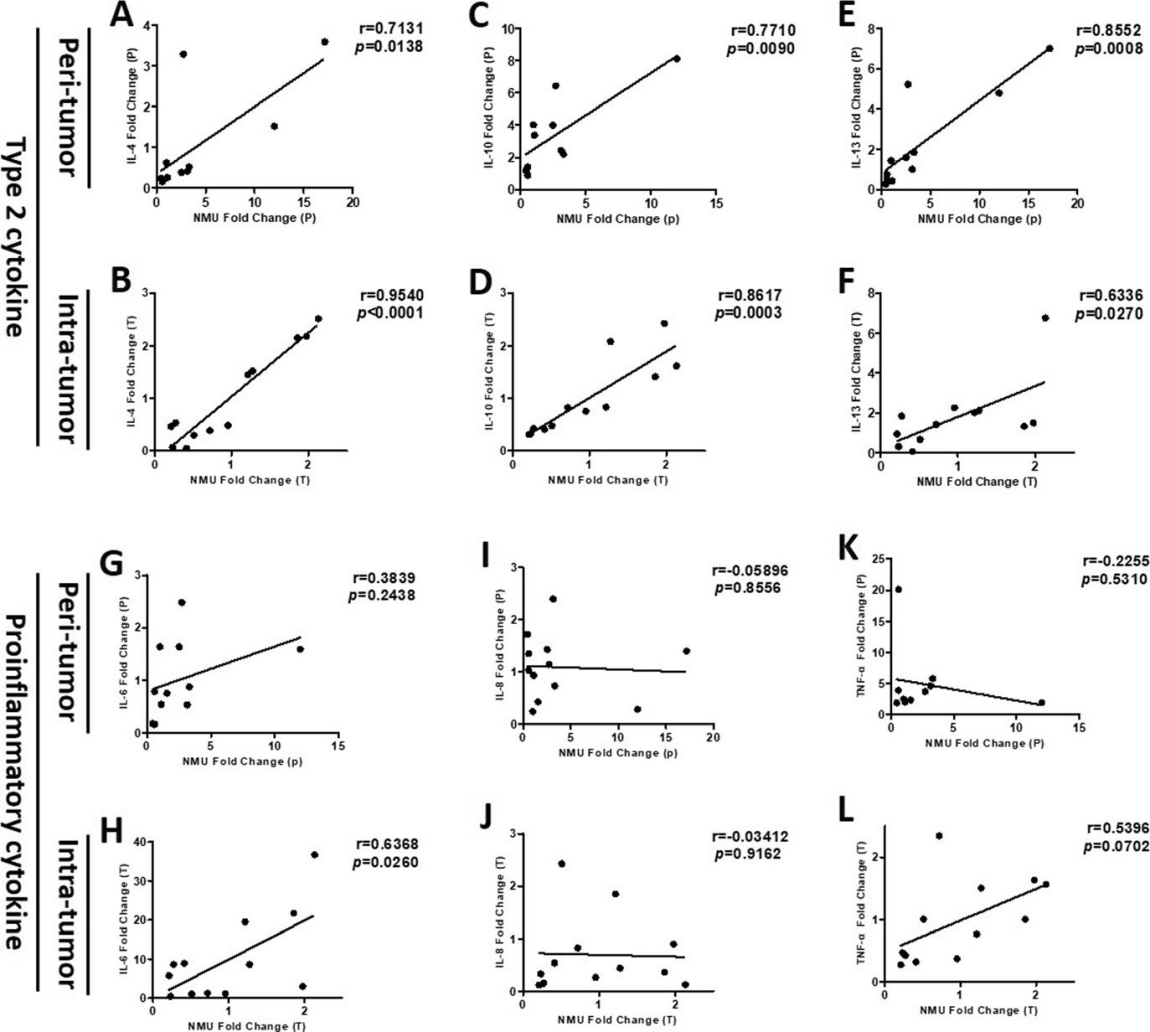
Correlation between the expression of NMU and inflammatory cytokines mRNA. NMU, IL-4, IL-10, IL-13, IL-6, IL-8, and TNF-α mRNA expression in 12 HCC peri- and intra-tumor tissue by using RT-qPCR. **a-f** The correlation between the expression of NMU and type 2 cytokines mRNA, such as IL-4, IL-10 and IL-13. (G-L) The correlation between the expression of NMU and proinflammatory cytokines mRNA, such as IL-6, IL-8 and TNF-α. NMU, neuromedin U; IL, interleukin; TNF-α, tumor necrosis factor-α; P, peri-tumor; T, intra-tumor

Furthermore, using RT-qPCR we examined NMU mRNA expression and the expression of various inflammatory cytokines in HCC peri- and intra-tumor tissue. Our results indicated that the expression of NMU mRNA in HCC peri- and intra-tumor tissue was positively correlated with the expression of type 2 cytokine mRNA, such as IL-4 (peri-tumor: r = 0.7131, *p* = 0.0138, intra-tumor: r = 0.9540, *p <* 0.001), IL-10 (peri-tumor: r = 0.7710, *p* = 0.0090, intra-tumor: r = 0.8617, *p* = 0.0003), and IL-13 (peri-tumor: r = 0.8552, *p* = 0.0008, intra-tumor: r = 0.6336, *p* = 0.0270) (Fig. 4a-f). There was no obvious correlation between NMU mRNA expression and the expression of proinflammatory cytokine mRNA in HCC peri- and intra-tumor tissue: IL-6 (peri-tumor: r = 0.3839, *p* = 0.2438), IL-8 (peri-tumor: r = − 0.05896, *p* = 0.8556, intra-tumor: r = − 0.03412, *p* = 0.9162), and TNF-α (peri-tumor: r = − 0.2255, *p* = 0.5310, intra-tumor: r = 0.5396, *p* = 0.0702) (Fig. 4g-l).

## Discussion

Studies have shown that overexpression of NMU is associated with tumorigenesis and development of various malignancies [12–19]. However, to date no studies have examined the association of NMU and HCC. Teranishi et al. [20] found that NMU mRNA was not detectable in the normal mouse liver, but its expression was significantly increased in the livers of NASH mice, and overproduction of NMU exacerbated the pathogenesis of NASH.

Our results showed that NMU protein was significantly elevated in the serum of HCC patients. Then, using immunohistochemical staining we examined the expression of NMU in peri- and intra-tumor HCC tissue. In other cancers, high cytoplasmic NMU protein expression is present in tumor cells. However, we found that NMU expression was located in intercellular space of HCC peri- and intra-tumor tissue, rather than hepatocytes or liver tumor cells. Our results, however, are similar to those of Teranishi et al. [20] who reported that NMU was distributed in the macrophage population in the livers of mice with NASH, consistent with the distribution of macrophages in the liver. In addition, Maiko Moriyama et al. [26] also found that NMU was expressed in wild type peritoneal macrophages. Ketterer et al. [13] also found moderate to strong immunoreactivity of NMU in enlarged intrapancreatic nerves of pancreatic cancer.

We then examined the prognostic value of NMU expression for HCC patients who received hepatectomy. Our results showed that the prognosis of HCC patients with high NMU expression in peri-tumor tissue was significantly poorer than those with low NMU expression, while the level of NMU expression in HCC tissue did not influence the prognosis of patients with HCC. Further study of the relationship between NMU expression and 16 clinicopathological features only found that high expression of NMU in HCC peri-tumor tissue was significantly correlated to a high level of serum AST. Subsequent univariate and Cox proportional hazard regression model analysis showed that high expression of NMU in peri-tumor tissue was a significant prognostic factor for OS and DFS of HCC patients after liver resection, as were tumor number, tumor size, major vascular invasion, Edmondson grade, TNM stage, Child-Pugh class, PLT count, and ALT and AST level. Furthermore, multivariate analysis indicated that NMU expression in peri-tumor tissue was a significant independent prognostic factor for OS. However, the level of intra-tumor tissue NMU expression did not predict the outcomes of patients with HCC.

Previous studies indicated that NMU could induce activation of group 2 innate lymphoid cells (ILC2s), resulting in the production of type-2 inflammatory cytokines [6, 7, 25]. M2 macrophages, which are important tumor-associated macrophages (TAM), play critical roles in tumor immune suppression, and are associated with a poor prognosis in numerous malignancies, including HCC [23, 27, 28]. Teranishi et al. [20] reported that NMU immunostaining was located in the macrophage population of the livers of NASH mice. Their results are consistent with our results that NMU is present in the stroma of peri- and intra-tumor tissue. Thus, we hypothesized that the role of NMU in HCC progression is related to M2 macrophages and type-2 inflammatory cytokines. In this study, we found that there was significant positive correlation between NMU expression level and M2 macrophage percentage (CD206/CD68) in HCC peri- and intra-tumor tissue. M2 macrophages express high levels of IL-4, IL-10, and IL-13, and low levels of proinflammatory cytokines IL-6, IL-8, and TNF-α [27, 29]. Our results demonstrated that the level of NMU mRNA in HCC peri- and intra-tumor tissue was positively related to the levels of type-2 cytokine mRNA, including IL-4, IL-10, and IL-13. However, we did not find a correlation between NMU and proinflammatory cytokines, such as IL-6, IL-8, and TNF-α. So we can speculate that the effect of NMU on the development and prognosis of HCC is closely correlated with M2 macrophages and the type-2 cytokine response.

A study by Yeung et al. [23] found that M2 macrophages in peri-tumor, but not in intra-tumor locations, significantly contribute to HCC progression. Their finding is consistent with our result that peri-tumor tissue, but not intra-tumor tissue NMU expression was significantly correlated with a poor prognosis.

There are limitations to this study. The number of patients was relatively small, and the study was a retrospective analysis. Our data set cannot be divided into a training data set and a test data set for statistical validation because of the small number of patients. Next, we are going to explore which cells secrete NMU, and the mechanisms by which NMU activate M2 macrophages or promote the expression of type-2 cytokines.

## Conclusions

NMU expression in peri-tumor tissue can be considered a prognostic biomarker for patients with HCC after curative hepatectomy. The promotion of HCC by NMU may be related to the activation of M2 macrophages and the secretion of type-2 inflammatory cytokines.

## Data Availability

The datasets used and/or analyzed during the current study available from the corresponding author on reasonable request.

## Abbreviations

AFP: Alpha-fetoprotein
AJCC: The American Joint Committee on Cancer
ALT: Alanine aminotransferase
AST: Aspartate aminotransferase
cDNA: Complementary DNA
CI: Confidence interval
CT: Computed tomography
DFS: Disease-free survival
ELISA: Enzyme-linked immunosorbent assay
HBsAg: Hepatitis B surface antigen
HBV: Hepatitis B virus
HCC: Hepatocellular carcinoma
HCV: Hepatitis C virus
HH: Hepatic hemangioma
HR: Hazard ratio
IHC: Immunohistochemistry
IL: Interleukin
ILC2s: Group 2 innate lymphoid cells
MRI: Magnetic resonance imaging
NASH: Non-alcoholic steatohepatitis
NMU: Neuromedin U
OS: Overall survival
PLT: Platelet
RFA: Radiofrequency ablation
RT-qPCR: Quantitative real-time PCR
TACE: Transcatheter arterial chemoembolization
TAM: Tumor-associated macrophages
TBIL: Total bilirubin
TNM: Tumor-node- metastasis
